# Anti-Obesity and Lipid Lowering Activity of Bauhiniastatin-1 is Mediated Through PPAR-γ/AMPK Expressions in Diet-Induced Obese Rat Model

**DOI:** 10.3389/fphar.2021.704074

**Published:** 2021-07-22

**Authors:** Reddy Sankaran Karunakaran, Oruganti Lokanatha, Ganjayi Muni Swamy, Chintha Venkataramaiah, Muppuru Muni Kesavulu, Chippada Appa Rao, Kameswara Rao Badri, Meriga Balaji

**Affiliations:** ^1^ Division of Cell Culture and Molecular Biology, Department of Biochemistry, Sri Venkateswara University, Tirupati, India; ^2^ Division of Molecular Biology, Department of Zoology, Sri Venkateswara University, Tirupati, India; ^3^ Department of Basic Sciences and Humanities, Sree Vidyanikethan Engineering College, Tirupati, India; ^4^ Department of Pharmacology and Toxicology, Cardiovascular Research Institute, Morehouse School of Medicine, Atlanta, GA, United States

**Keywords:** 3T3-L1 cells, adipogenesis, AMPK, bauhiniastatin-1, diet-induced obesity, insulin resistance, molecular docking, PPAR-γ

## Abstract

**Objective:** To evaluate the therapeutic efficacy and underlying molecular mechanisms of Bauhiniastatin-1 (BSTN1) to alleviate adiposity in diet-induced obese rodent model and in 3T3-L1 cells.

**Methods:** BSTN1 was purified and confirmed through HPLC. *In-vitro* experiments such as MTT assay, Oil Red-O (ORO) stain, cellular lipid content, glycerol release and RT-PCR analysis were performed in 3T3-L1 cells in the presence and absence of BSTN1. In animal experiments, rats were divided into Group-I: normal pellet diet-fed, Group-II: HFD-fed, Groups-III, IV and V: HFD-fed BSTN1 (1.25, 2.5, and 5 mg/kg.b.wt./day/rat)-treated and Group-VI: HFD-fed Orlistat-treated. The rats were fed either normal diet or high fat diet (HFD) for 18 weeks and water *ad-libitum*. BSTN1 was orally administered from 13th week onwards to the selected HFD-fed groups. Body composition parameters, biochemical assays, histopathology examination and western blot analysis were performed to identify the predicted targets related to obesity. Molecular docking studies threw light on the binding interactions of BSTN1 against PPAR-γ, FAS and AMPK.

**Results:** BSTN1 at 20 μM significantly (*p* < 0.001) inhibited adipocyte differentiation and lipid accumulation in 3T3-L1 cells. A conspicuous down-regulation in the mRNA expression levels of PPAR-γ, FAS and SREBP1 was observed but AMPK expression remained unchanged in BSTN1 treated 3T3-L1 cells. A substantial decrease in body weight gain, fat percent, total body fat, serum and liver lipid profile (except high-density lipoprotein), glucose, insulin and insulin resistance in BSTN1 treated rats was noticed in a dose dependent manner. In BSTN1 (5 mg/kg.b.wt.)-treated groups significantly (*p* < 0.01) elevated plasma adiponectin level but reduced leptin level as well as fall in serum AST and ALT were noticed. Further, the disturbed structural integrity and architecture of adipose and hepatic tissues due to high fat diet feeding were considerably recovered with BSTN1 treatment. Down-regulation in the protein expression level of PPAR-γ and activation of AMPK through phosphorylation was observed in BSTN1 treated rats than the untreated. Molecular docking studies revealed strong binding interactions of BSTN1 against PPAR-γ and AMPK and thus supported the experimental results.

**Conclusion:** Taken together, the results suggest that BSTN1 could be a promising pharmacological molecule in the treatment of obesity and dyslipidemia.

## Introduction

The epidemic of obesity has become a public health issue in both developed and developing countries as it strongly predisposes to type-2 diabetes, dyslipidemia, hypertension, cardiovascular diseases (CVDs), non-alcoholic fatty liver disease (NAFLD), sleep apnea, infertility and certain types of cancers (Hurt et al., 2010). The excess calories consumed than expended gets accumulated as fats (triglycerides) in adipocytes and leads to growth and expansion of white adipose tissue (WAT) by the process of hypertrophy and hyperplasia (Meln et al., 2019). At cellular level, adipogenesis is a complex process involving preadipocytes clonal expansion, differentiation and intracellular lipid accumulation which is tightly regulated at transcriptional level by peroxisome proliferator activated receptor-γ (PPAR-γ), members of CCAAT/enhancer binding protein (C/EBP) family and sterol regulatory element binding protein (SREBPs) (Moseti et al., 2016; Chang and Kim 2019).

White adipose tissue not only serves to store excess fats but also performs vital endocrine functions such as secretion of adipokines and release of inflammatory cytokines that play prevailing role in disturbing general energy homeostasis and insulin sensitivity resulting in various metabolic disorders (Longo et al., 2019). Similarly, due to excess accumulation of fats in hepatic tissue in conditions like hepatic steatosis, non-alcoholic steatohepatitis (NASH) and NAFLD the metabolic functions of liver are derailed (Nassir et al., 2015).

Various therapeutic approaches such as lifestyle modification, pharmacotherapy, and bariatric surgery are advocated to combat obesity ailments (Apovian et al., 2015). Although lifestyle modification is the cornerstone of weight management, it is difficult to put it in practice in day-to-day life and to sustain. On the other hand, bariatric surgery involves high risk and high cost as well. In view of this, pharmacotherapy is typically considered as an effective treatment option. Therapeutic molecules that work as lipase inhibitors, appetite suppressors, antilipidemic agents and inhibitors of adipogenesis are being intensely researched to develop effective drugs to treat obesity ailments (Balaji et al., 2016; Liu et al., 2020). Considering the growing public inclination toward natural product-based therapeutics, the present work was carried out with Bauhuniastatin-1 isolated from *Bauhinia purpurea*.

*Bauhinia purpurea* Linn. is a fast-growing medium sized flowering plant of Fabaceae family, widely grown in south-east Asian countries including India and China. This plant species is rich in phytochemicals like bauhiniastatins, isoquercitin, lutein, β-sitosterol, pacharin, astragalin, stigmasterol, kaempferol, lupeol etc. Its leaves, flowers and seeds are eaten along with rice in some parts of South East Asian countries (Kumar and Chandrashekar 2011). *B. purpurea* possesses anti-diabetic, anti-inflammatory, antimicrobial, analgesic and anti-neoplastic activities and has been used in traditional and folk medicine (Zakaria et al., 2007). In our previous study, we reported the anti-hyperlipidemic activity with crude extract of *B. purpurea.* (Padmaja et al., 2014).

In the present study, we isolated and purified the active principle, Bauhiniastatin-1, (BSTN1) elucidated its anti-obesity activity and underlying molecular mechanisms using diet-induced obese rat model. Adipogenic, lipogenic and lipolytic studies are complemented using the most widely used 3T3-L1 preadipocytes. In addition, molecular docking studies were carried out to understand the mechanisms of action of BSTN1.

## Materials and Methods

### Chemicals and Materials

Dulbecco’s Modified Eagle’s Medium (DMEM), fetal bovine serum (FBS), 3-isobutyl-1-methyl-xanthine (IBMX), penicillin and streptomycin, isopropanol, insulin, and dexamethasone (DEXA) were procured from Thermo Fisher Scientific (Berkeley, MO, United States). MTT (3-(4, 5-dimethylthiazol-2-yl)-2, 5-diphenyltetrazolium bromide) and Oil-Red-O (ORO) stain were procured from Sigma Aldrich, (St Louis, MO, United States). Other solvents, chemicals and reagents utilized for the experiments were of analytical grade.

### Isolation and Purification of Bauhiniastatin-1

The bark of *B. purpurea* was collected from the Seshachalam forests, spread around Tirupati, Andhra Pradesh, India. It is authenticated by taxonomist in the department of Botany, Sri Venkateswara University, Tirupati, voucher number is 136 and specimen was preserved in departmental herbarium. The bark of *B. purpurea* was powdered, extracted with ethanol and further fractionated by column chromatography using different solvents (Padmaja et al., 2014). The collected fractions were subjected to LC-MS analysis on 6520 Accurate Q-TOF (Agilent Technologies, Inc. Santa Clara, CA, United States) mass spectrometer to identify the major compounds. Bauhiniastatin-1 was purified using an HPLC system equipped with a binary gradient system, a variable UV-VIS-detector and a Rheodyne Model 7725 injector with a loop size of 20 μl, and an integrator. Reverse phase chromatographic analysis was carried out in isocratic conditions using a C-18 reverse phase column (250 × 4.6 mm id., 5 μl C-18) at 40°C. Mobile phase consisted of methanol:water (20:80 v/v) with a flow rate of 1 ml/min. The detection of compounds was performed at 220 nm. A single sharp peak at 5.942 min of retention time was identified as BSTN1 (Pettit et al., 2006).

### Cell Culture and Differentiation of Adipocytes

The 3T3-L1 pre-adipocytes of American Type Culture Collection (ATCC) cells were maintained and cultured in DMEM supplemented with 10% FBS at 37°C in a humidified atmosphere with 5% CO_2_. The 3T3-L1 were grown to confluence, stimulated with adipogenesis differentiation medium of induction (DMI) consisting of DMEM, 10% FBS, 0.5 mM IBMX, 1 μM DEXA and 10 μg/ml insulin for 2 days followed by treating cells with differentiation medium (DM) (DMEM with 10% FBS and 10 μg/ml insulin) for additional 8–10 days (Badri et al., 2008). All the media that we used contained 100 IU/ml penicillin and 100 mg/ml streptomycin. A volume of 0.01% DMSO was used as vehicle control for *in-vitro* experiments. For evaluating anti-adipogenic effects of BSTN1, 3T3-L1 cells cultured and adipogenesis was induced by DMI in 12 well plates and treated with different concentrations of BSTN1 (5, 10, and 20 μM) in DM. The adipocytes were stained for neutral lipids (lipid droplets) and observed under a bright field microscope or used for other studies.

### Cell Viability Assay (MTT Assay)

The 3T3-L1 preadipocytes were cultured in DMEM and cell viability assays were conducted as previously described (Lee et al., 2018).

### Oil Red O Staining: Determination of Lipid Content

Lipid contents in adipocytes were visualized as well as measured using Oil Red-O (ORO) staining (Nunez et al., 2019).

### Lipolysis Studies

The lipolysis studies were conducted by measuring glycerol levels released into the cell culture medium, using commercial kit (Lipolysis assay kit, ab185433, Abcam, and Shanghai) following manufacturer’s instructions (Shobha et al., 2017). The glycerol content was expressed as nmol/well.

### RT-PCR Studies: mRNA Expression

Total RNA was isolated from 3T3-L1 cells by using tri-reagent (Sigma Aldrich, Bangalore India) according to manufacturer’s protocol and reverse transcribed to obtain cDNA using cDNA synthesis kit (Applied Bio Systems, Foster City, CA, United States) (Galateanu et al., 2012). About 20 ng of cDNA was used for semi-quantitative RT-PCR (BioRad CFX96, Real-Time PCR) using SYBR green master mix and the conditions were kept as follows: 95°C for 10 min, followed by 36 cycles for 30 s at 72°C and 1 min at 60°C and followed by extension for 15 s at 72°C. Data was analyzed using ΔΔCt method and values were expressed in terms of relative fold change (RFC). PCR reactions were run in triplicate for each sample, and transcription levels of every gene were normalized to the level of β-actin. The PCR amplification was performed with transcript specific primers (Supplementary Table 1).

### Animal Studies

Male WNIN rats (aged 5–6 weeks), normal pellet diet and high fat diet were obtained from National Institute of Nutrition (NIN), Hyderabad, India. After one-week acclimatization, rats were maintained at standard laboratory conditions (temperature: 23 ± 2°C; humidity: 40–60%), fed with either normal diet or HFD and water ad-libitum for 18 weeks as described in experimental design (Donovan et al., 2009). The composition of normal pellet diet and HFD (fat-60%) are given in supplementary files (Supplementary Tables 2, 3). To test for therapeutic activity, different concentrations of BSTN1 (1.25, 2.5, and 5 mg/kg b. wt.) suspended in 0.5% carboxymethylcellulose (CMC) were orally administered for 6 weeks from 13th week onwards using an intra-gastric tube. We selected these concentrations based on our initial pilot studies using solvent extracts (Padmaja et al., 2014). Guidelines of IAEC (No:55/2012/(i)/a/CPCSEA/IAEC/SVU/MBJ, dated: 8-7–2012) were followed to conduct the animal experiments.

### Experimental Design

Rats initially weighing 160–180 g were randomly divided into six groups of six each (*n* = 6).

Group I: Normal pellet diet fed rats
Group II: HFD-fed rats (Placebo)
Group III: HFD fed + BSTN1 (1.25 mg/kg b. wt./day) treated rats
Group IV: HFD fed + BSTN1 (2.5 mg/kg b. wt./day) treated rats
Group V: HFD fed + BSTN1 (5 mg/kg b. wt./day) treated rats
Group VI: HFD fed + Orlistat (5 mg/kg b. wt./day) treated rats

### Measurement of Body Composition Parameters, Food and Water Intake

Body weight, lean body mass, fat percent, fat free mass of each rat was measured by Total Body Electrical Conductivity (TOBEC) using a small animal body composition analysis system (EM-SCAN, Model SA-3000 Multi detector, Springfield, United States). Food intake, water intake and behavior was monitored every day. At the end of the experiment, animals were anesthetized using isoflurane, blood was collected by heart puncture method. Plasma and/or serum were separated by centrifugation at 2,500 rpm for 15 min. Various organs and tissues including abdominal adipose tissue and liver were dissected, and stored appropriately. For histology studies, tissues were fixed and processed as described in later sections.

### Estimation of FBG, PPBG, F-Ins and HOMA-IR

After overnight fasting of experimental rats, the glycemic parameters such as fasting blood glucose (FBG) and after food intake post-prandial blood glucose (PPBG) were monitored by ACCU-Check, Glucometer. Serum fasting-insulin (F-Ins) was measured using commercially available enzyme-linked immunosorbent (ELISA) kit (Elabscience, United States) and homeostasis model assessment index for insulin resistance (HOMA-IR) was used to identify insulin resistance as follows:

HOMA-IR = [fasting glucose (mg/dl) X fasting insulin (μIU/ml)]/405.

### Plasma Leptin and Adiponectin Levels

Plasma leptin and adiponectin are key adipokines secreted by adipocytes. Adipokine levels were measured in experimental rats using enzyme-linked immunosorbent assay kits (Crystal Chem, Downers Grove, IL, United States). These assays were performed in duplicates (*n* = 6), as per the manufacturer’s guidelines and adipokine levels were expressed in ng/mL (Margoni et al., 2011).

### Estimation of Serum Lipid Profile

Serum total cholesterol (TC) was estimated by CHOD-PAP method, triglycerides (TGs) and HDL-cholesterol were estimated by GPO-TOPS method, VLDL-cholesterol, LDL-cholesterol were estimated by selective inhibition method (Agappe Diagnostics Ltd., Kerala, India). The phospholipids (PLs) and free fatty acids (FFAs) were assessed by using quantification assay kits, Sigma Aldrich, Bangalore, India.

### Estimation of Hepatic Lipid Levels

Lipids were extracted from the liver tissues of experimental animals (Floch et al., 1957). In brief, the tissues were rinsed with ice-cold physiological saline, homogenized in cold chloroform-methanol (2:1, v/v) and the contents were extracted for 24 h. The extraction was repeated four times. The combined filtrate was washed with 0.7% potassium chloride and the aqueous layer was discarded. The organic layer was made up to a known volume with chloroform and used for hepatic lipid analysis.

### Measurement of AST and ALT Activities

Hepatic marker enzymes, aspartate transaminase (AST) and alanine transaminase (ALT) activities were estimated at the end of the experiment by using commercially available kit (Agappe Diagnostics Ltd., Kerala, India) following the manufacturer's protocol.

### Western Blot Analysis

Adipose and hepatic tissue proteins were washed with sterile 1X PBS and homogenized in RIPA lysis buffer (Product No. R0278, Sigma Aldrich, Bangalore, India) containing protease inhibitors (PI) (RIPA + PI: 1 ml + 40 µl), centrifuged (14,000 rpm for 10 min at a 4°C) and the supernatant was collected. The protein concentrations were measured using the Bradford method. Forty µg of proteins were resolved on 10% SDS-PAGE gel and transferred onto a PVDF membrane. To block non-specific binding sites, blots were incubated at room temperature with 5% skimmed milk (w/v) for 1 h on shaking rocker followed by overnight incubation with specific primary antibodies of anti-PPAR-γ (Catalog No. C26H12, ILS-CST, India), anti-AMPK (Catalog No. A17290)/phospho-AMPK (Catalog No. AP0116) and mouse anti-β actin (Catalog No. AC004) (Abclonal Technology, United States) at 4°C. The immuno-reactive antigen was then recognized by incubation with HRP-conjugated secondary antibody (Abclonal Technology, United States) for 1 h at RT. After washing with 1X TBST, the membrane was treated with chemiluminescent ECL detection reagent (1:1) (Bio-Rad) and the immuno-reactive bands were visualized by ChemiScope western blot imaging system (Make: Wipro GE Healthcare) (Lira et al., 2010).

### Histopathological Examination

Adipose and liver tissues were collected from both control and experimental rats and fixed in formalin solution. A small piece of tissue was cut, trimmed, processed and prepared paraffin blocks. Then the paraffin blocks were sectioned (5–8 µm) using microtome and stained using haematoxylin and eosin (H&E) following standard histology protocol (Badri et al., 2013).

### Molecular Docking Studies (Accession of Target Protein)

The three-dimensional structure of FAS (6NNA), AMPK (6C9F), PPAR-γ (3WMH), and BSTN1 were downloaded from the RCSB protein Data Bank and Pub chem. The atomic coordinates of the ligand were geometrically optimized using Argus Lab 4.0.1. *In-silico* studies were carried against FAS (6NNA), AMPK (6C9F), and PPAR-γ (3WMH), with ligand (BSTN1) using the docking program Patchdock (Xiao et al., 2013). After the docking, protein–ligand complexes were studied using PyMol viewer tool (www.pymol.org)1. Protein and ligand interactions were analyzed and visualized through PyMol viewer tool (www.pymol.org)1.

### Statistical Analysis

The results are expressed as the mean ± standard deviation (SD), and comparison was made by using one-way ANOVA program followed by Tukey’s post hoc tests to study the significance level (SPSS, version 17.0; SPSS Inc., Chicago, IL, United States).

## Results

### LC-MS and HPLC Analysis

**FIGURE 1:**
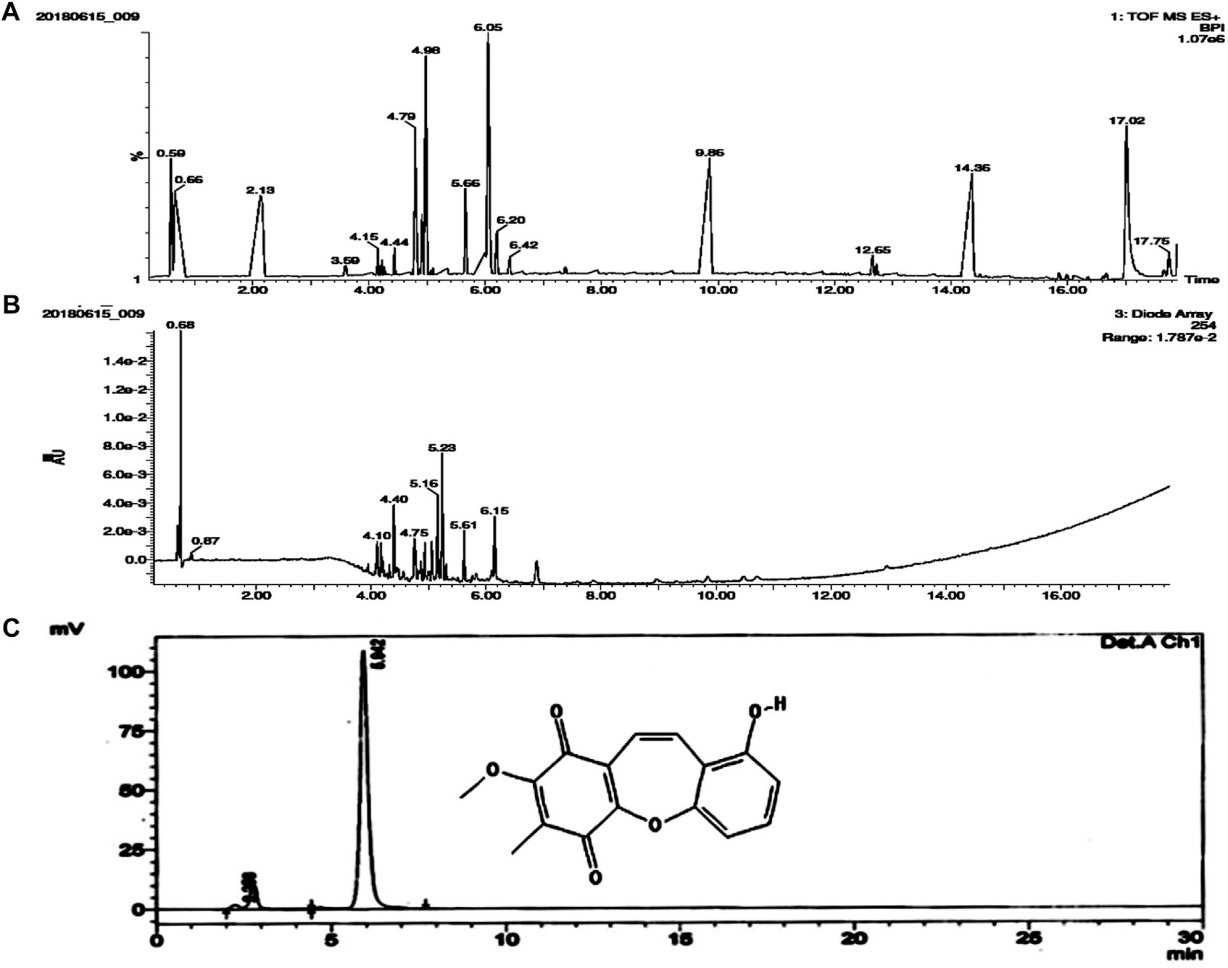
Molecular characterization of isolated compound/s from the chloroform fraction of *B. purpurea* ethanolic extract. **(A)** and **(B)** LC-MS analysis showing TOF MS ES + BPI and Diode Array (Retention Time) **(C)** HPLC peak representing structure and retention time (5.942) of BSTN1.

The chloroform fraction of ethanolic extract of *B. purpurea* was subjected to LC-MS/MS analysis. Seventeen compounds were identified from which BSTN1 (m.wt.: 283.0496) was isolated, purified and confirmed by HPLC (Figures 1A–C).

### Cell Viability Studies by MTT Assay

**FIGURE 2:**
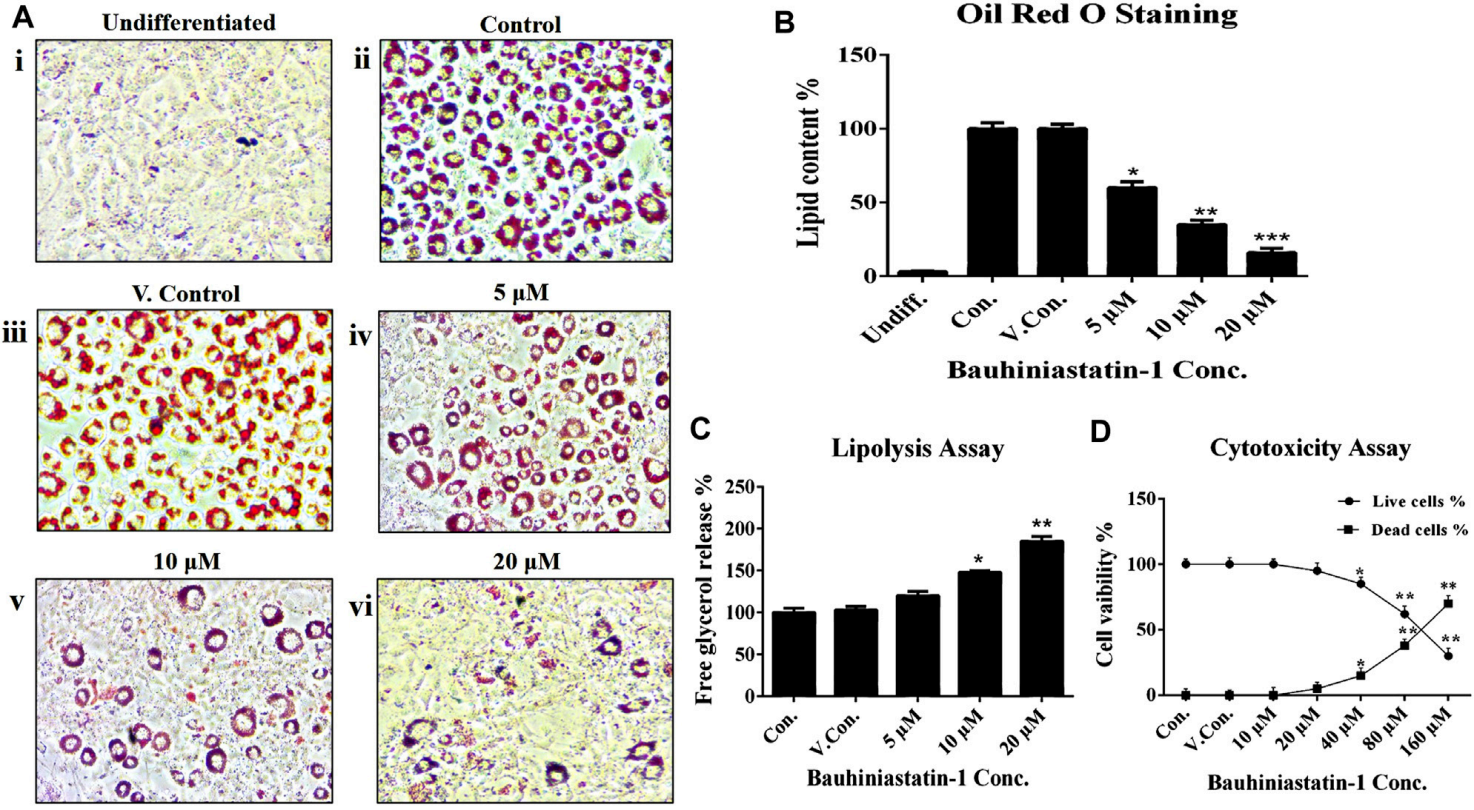
Effect of BSTN1 on lipid levels in 3T3-L1 cells by **(A)** Oil Red O staining of 3T3-L1 cells (Magnification 40×). (i) Undifferentiated, untreated pre-adipocytes (ii) Differentiated, untreated adipocytes (control) (iii) DMSO (0.01%) treated adipocytes (vehicle control). (iv), (v), and (vi) BSTN1 treated adipocytes (5, 10, and 20 μM) showing decreased adipogenesis in dose dependent manner. **(B)** Quantification of lipid content (%) by Oil Red O stain extraction in BSTN1-treated and other groups. **(C)** Lipolysis assay indicating the amount of glycerol content released from 3T3-L1 adipocytes into the media. **(D)** Cytotoxicity assay results on the percentage of cell viability of untreated, DMSO and BSTN1 treated pre-adipocytes determined after 48 h using MTT assay. Data are presented as mean ± SD of triplicate. *, **, and *** indicate significant difference between control and treated 3T3-L1 cells at *p* < 0.05, *p* < 0.01, and *p* < 0.001 respectively.

The effect of BSTN1 on cell viability of 3T3-L1 cells and cytotoxicity was analyzed in the dose range of 10–160 μM, at 48 h using MTT assay. The BSTN1 showed IC50 value of 118 µM (Figure 2D).

### Effect of BSTN1 on Adipogenesis and Lipid Content

The BSTN1 at a dose of 20 µM could markedly reduce adipogenesis and intracellular lipid levels in 3T3-L1 cells, as observed by Oil-Red-O-stained images (Figure 2A). Furthermore, lipid content of adipocytes was measured by extracting (using isopropanol) the Oil Red O stain from 3T3-L1 adipocytes (Figure 2B). To complement these studies, we verified the lipolytic capacity of BSTN1 by quantifying the levels of glycerol release from treated vs. control and vehicle control 3T3-L1 adipocytes. Significantly (*p* < 0.01) high level of glycerol release was observed in adipocytes treated with BSTN1 than untreated cells and the maximum lipolytic activity was noticed at a concentration of 20 µM (Figure 2C).

### Effect of BSTN1 on mRNA Expression

**FIGURE 3:**
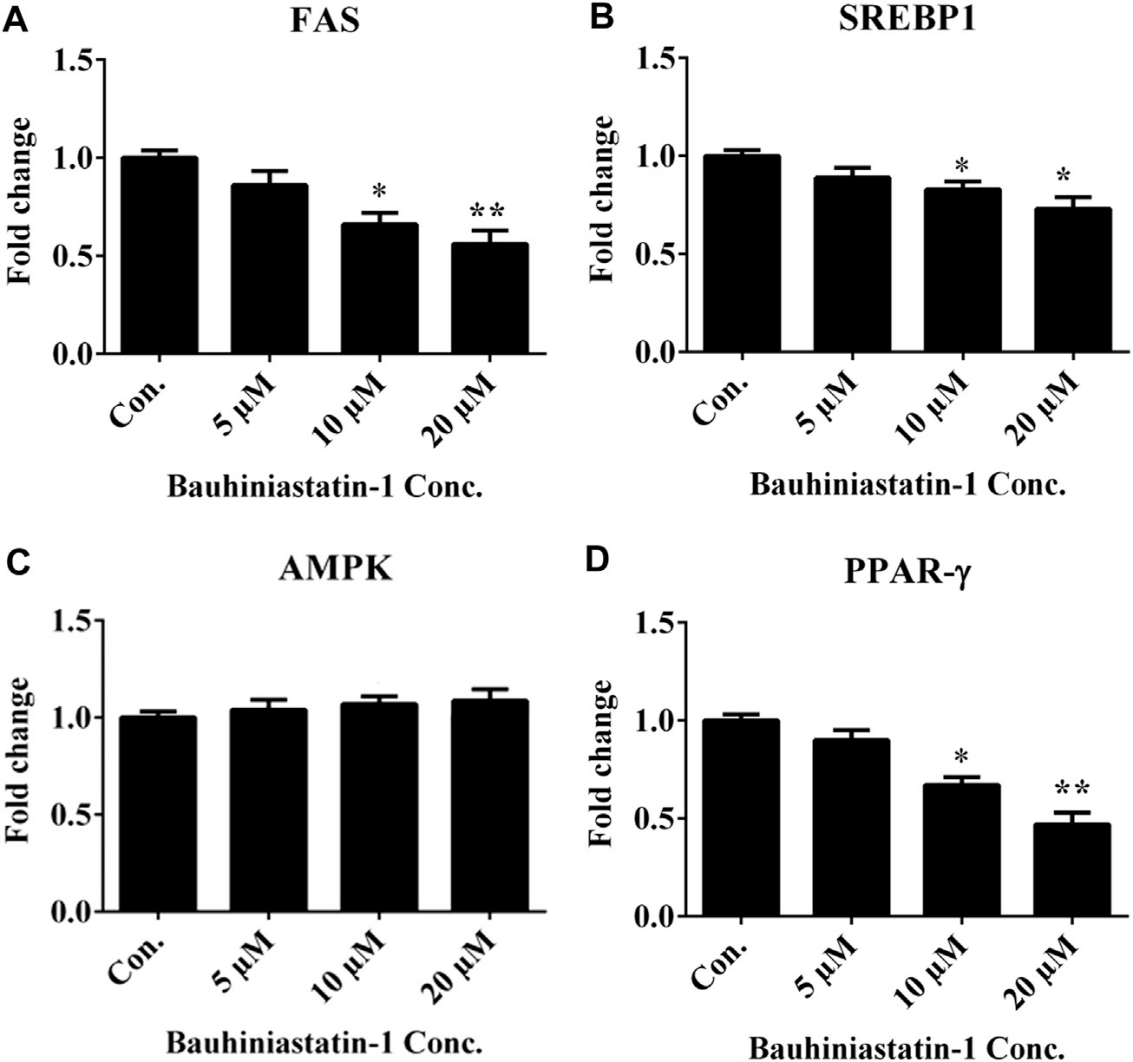
Effect of BSTN1 on mRNA expression of target genes in 3T3-L1 cells by RT-PCR. Quantitation of fold change of key adipogenic and lipogenic markers **(A)** FAS mRNA **(B)** SREBP1 mRNA, **(C)** AMPK mRNA and **(D)** PPAR-γ mRNA. Data are presented as mean ± SD of triplicate. * and ** indicate significant difference between control and treated 3T3-L1 cells at *p* < 0.05 and *p* < 0.01, respectively. FAS, fatty acid synthase; SREBP1, sterol regulatory element-binding protein 1; AMPK, AMP-activated protein kinase and PPAR-γ, peroxisome proliferator-activated receptor-γ.

The mRNA expression levels of key adipogenic and lipogenic transcriptional factors, FAS, SREBP1, AMPK and PPAR-γ in differentiated 3T3-L1 adipocytes were estimated in the absence and presence of BSTN1 (5, 10, and 20 µM) (Figure 3). The expression of FAS, SREBP1, and PPAR-γ were down-regulated, while that of AMPK remained unchanged with increasing concentration of BSTN1 as represented in Figures 3A–D.

### Effect of BSTN1 on Protein Expression

**FIGURE 4:**
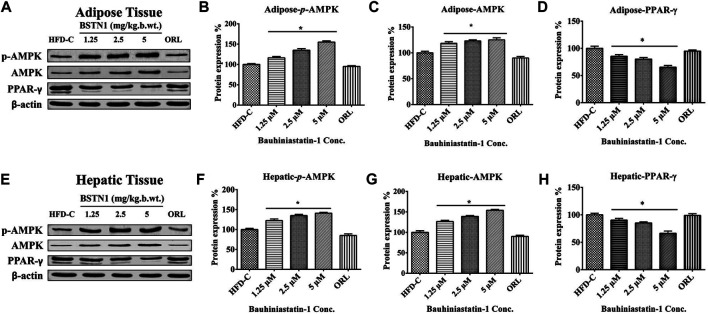
Western blot analysis: Protein expression of control and treated rats in **(A)** adipose tissue and **(E)** liver tissue. *β*-actin is used as a housekeeping gene. Histograms showing quantification of expressed proteins: **(B)** Adipose-*p*-AMPK **(C)** adipose-AMPK **(D)** adipose-PPAR-γ, **(F)** Hepatic-*p*-AMPK **(G)** Hepatic-AMPK and **(H)** Hepatic-PPAR-γ. Histograms represent mean ± SD, *n* = 6. * indicates significant difference (*p* < 0.01) between HFD control and treated groups. AMPK, AMP-activated protein kinase and PPAR-γ, peroxisome proliferator-activated receptor-γ.

Figure 4 shows the protein expression levels of AMPK/phosphor-AMPK and PPAR-γ in hepatic and adipose tissues of obese control and obese-treated rats. BSTN1 treatment induced activation of AMPK as evident from its phosphorylation, in a dose dependent manner. However, down-regulation of PPAR-γ with increasing concentration of BSTN1 was observed in treated rats. The results indicate the efforts of BSTN1 to maintain energy homeostasis and reduce the fat accumulation in liver and adipose tissues.

### Body Composition, Body Weight, Food and Water Intake of Obese Rats

Table 1 depicts the changes in body weight and fat percent of experimental rats. Consumption of HFD for 18 weeks resulted in significant rise in body weights (486 ± 9.0 g) and total body fat levels (79 ± 7.8 g) in HFD control group, compared to normal control group of rats whose body weight and total fat were 248.1 ± 6.3 g and 10.2 ± 1.4 g, respectively. Oral administration of BSTN1 considerably reduced body weight gain and body composition parameters in a dose dependent manner, with maximum reduction in body weight (320 ± 13.9 g) and total fat levels (24.9 ± 1.9 g) being noted in rats treated with 5 mg/kg b. wt. of BSTN1. However, no significant difference was observed in food and water intake (Table 1) of BSTN1 treated groups indicating that BSTN1 could reduce weight gain in obese rats without compromising food and water intake quantity. The behavior of all the animals was normal.

**TABLE 1:** Effect of BSTN1 on food intake, water intake, body weight and body composition parameters of treated and untreated rats.

Parameters	Normal control	HFD control	BSTN1-1.25 mg	BSTN1-2.5 mg	BSTN1-5 mg	ORL-5 mg
Food intake (g/day/rat)	15.7 ± 0.7	14.3 ± 1.1	14.5 ± 0.8	14.7 ± 0.1	15.2 ± 0.9	15.1 ± 0.3
Water intake (mL/day/rat)	21.4 ± 1.2	26.1 ± 0.8	24.5 ± 1.0	25.7 ± 0.7	25.9 ± 1.1	24.5 ± 0.5
Initial body weight (g)	168.3 ± 7.0	182.5 ± 9.4	177.3 ± 8.4	179.3 ± 10.7	183.2 ± 8.0	180.8 ± 5.9
Final body weight (g)	248.1 ± 6.3	486.3 ± 9.0^#^	445.2 ± 11.0^*^	401.5 ± 12.0^**^	320.5 ± 13.9^***^	312.7 ± 7.7^***^
Body weight gain (g)	79.8 ± 4.6	303.8 ± 15.5^#^	267.8 ± 10.7^*^	222.2 ± 14.8^**^	137.3 ± 17.8^***^	131.8 ± 10.7^***^
Lean body mass (g)	237.9 ± 7.0	407.3 ± 7.8^#^	382.6 ± 13.6^*^	358.6 ± 14.2^**^	295.7 ± 14.4^***^	290.1 ± 6.5^***^
Total body fat (g)	10.2 ± 1.4	79 ± 7.8^#^	62.5 ± 3.8^*^	42.9 ± 2.4^**^	24.9 ± 1.9^***^	22.6 ± 4.6^***^
Body fat (%)	4.1 ± 0.6	16.2 ± 1.5^#^	14.1 ± 1.1^*^	10.7 ± 0.9^**^	7.8 ± 0.7^***^	7.2 ± 1.4^***^
Fat free mass (g)	147.1 ± 4.1	225.4 ± 5.3^#^	215.5 ± 8.3^*^	206.8 ± 8.5^**^	175.9 ± 8.3^***^	173.3 ± 3.9^***^
Total body water	620.1 ± 18.5	972.6 ± 23.9^#^	928.2 ± 37.4^*^	888.9 ± 38.3^**^	749.7 ± 37.2^***^	738 ± 17.4^***^
Total body Na+	859.4 ± 25.7	1,349 ± 33.1^#^	1,287.3 ± 52.0^*^	1,232.7 ± 53.2^**^	1,039.4 ± 51.7^***^	1,023.2 ± 24.2^***^
Total body K+	1783.1 ± 51.0	2,752.4 ± 65.6^#^	2,630.3 ± 103.0^*^	2,522.3 ± 105.4^**^	2,139.5 ± 102.3^***^	2,107.3 ± 48.0^***^

The data is expressed as the mean ± SD, *n* = 6. ^#^ *p* < 0.001 indicates significant difference between HFD control (placebo) and normal control. *, ** and *** indicate significant difference between HFD control and treated groups at *p* < 0.05, *p* < 0.01, and *p* < 0.001, respectively.

### Effect of BSTN1 on Leptin and Adiponectin Levels and Adipose Tissue Architecture

**FIGURE 5:**
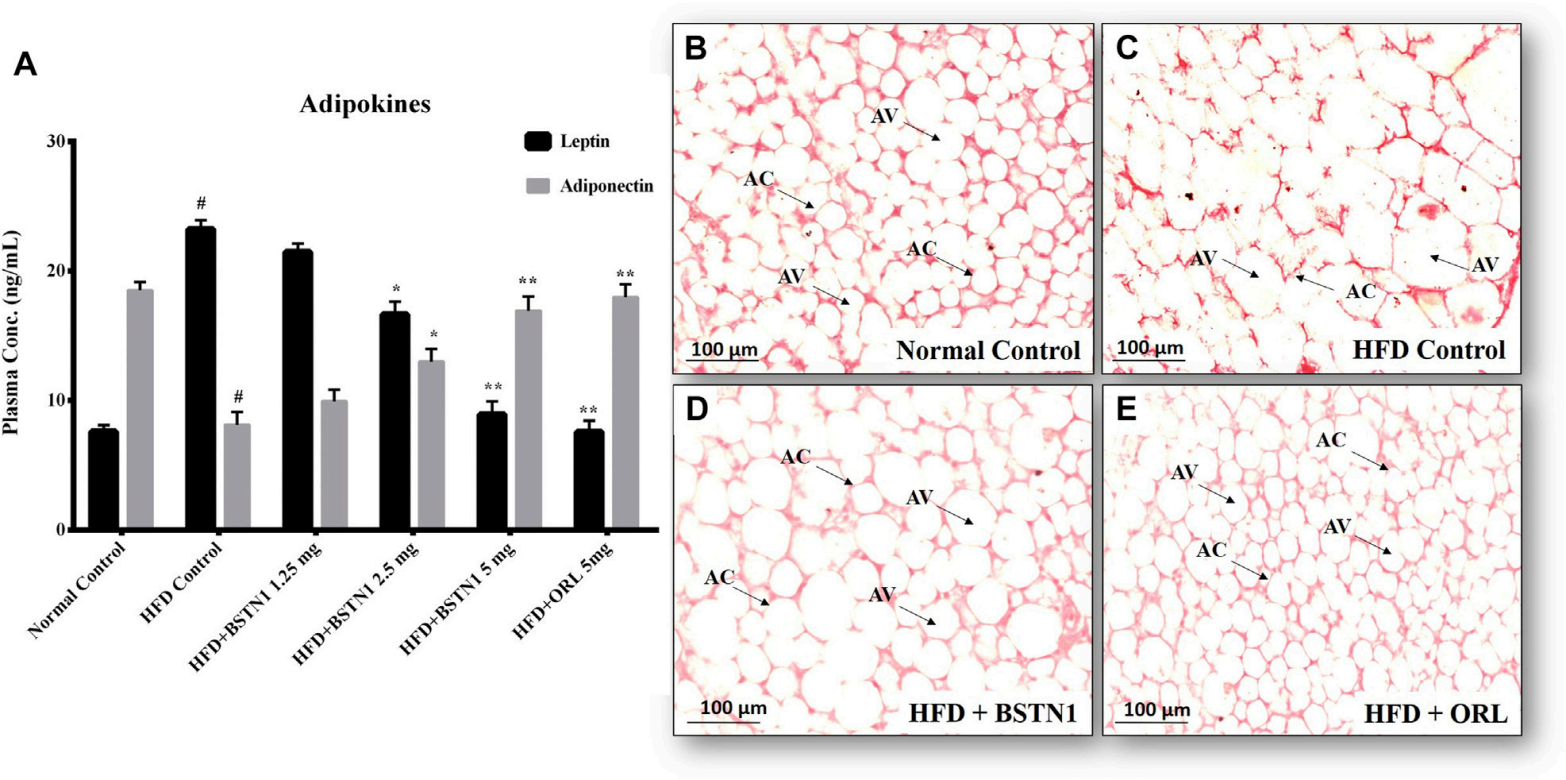
Effect of BSTN1 on adipokines and histology of white adipose tissue (40x magnification). **(A)** Plasma adipokines levels represented as mean ± SD, *n* = 6. ^#^ denotes significant (*p* < 0.001) difference between normal control and HFD control. * and ** denote significant difference between HFD control and treated groups at *p* < 0.05 and *p* < 0.01, respectively. **(B)** Histology of normal control adipose tissue **(C)** HFD-fed group showing enlarged and lipid laden adipocytes **(D)** HFD fed BSTN1 treated group showing reduction in adipocytes’ size and lipid content **(E)** HFD fed orlistat treated group. AC, Adipocyte Cell and AV, Adipocyte Volume. Scale bar: 100 µm.

Figure 5 shows the systemic levels of leptin and adiponectin in control and experimental obese rats. We observed markedly elevated levels of plasma leptin but decrease in adiponectin levels in HFD fed rats, when compared to the normal rats. Interestingly, treatment with BSTN1 has significantly (*p* < 0.01) decreased leptin levels, while the levels of adiponectin were increased. The H&E-stained adipose tissue sections of control rats showed the normal adipocyte structure (Figure 5B). In contrast, adipocytes of HFD-induced obese rats are significantly larger (Figure 5C) (hypertrophy) with indistinct cell walls compared to adipocytes of control rats. Relatively smaller sized adipocytes were visualized in BSTN1 treated rats indicating reduced lipid droplets, and this effect was more significant than even orlistat treated group (Figures 5D,E).

### Effect of BSTN1 on Hepatic Enzymes (AST and ALT) and Hepatic Tissue Architecture

**FIGURE 6:**
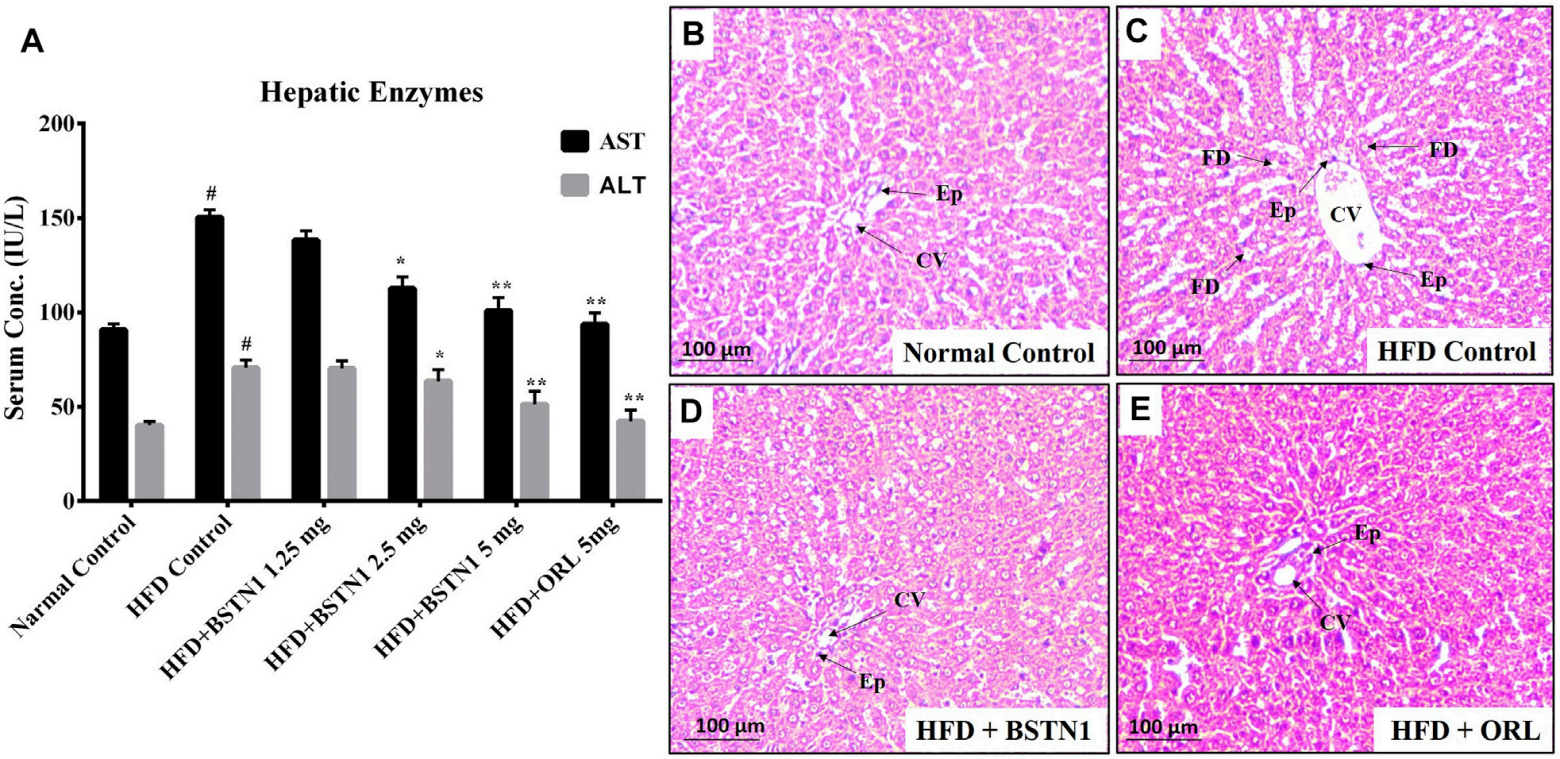
Effect of BSTN1 on liver enzymes (AST and ALT) levels and liver tissue histology (40x magnification). **(A)** Serum AST and ALT levels represented as mean ± SD, *n* = 6. ^#^ denotes significant (*p* < 0.001) difference between normal control and HFD control. * and ** denote significant difference between HFD control and treated groups at *p* < 0.05 and *p* < 0.01, respectively. **(B)** Histology of normal control hepatic tissue **(C)** HFD fed group showing disrupted tissue structure with enlarged central vein **(D)** HFD fed BSTN1 treated group showing improvement in hepatic structure **(E)** HFD fed orlistat treated group. CV, Central Vein; FD, Fat Droplets and Ep, Epithelial cells. Scale bar: 100 µm.

Figure 6 depicts the activities of aspartate transaminase (AST) and alanine transaminase (ALT) in control and experimental obese rats. Administration of BSTN1 significantly (*p* < 0.01) reduced AST and ALT levels in dose dependent manner when compared to HFD control rats. Hepatic tissues of untreated and treated rats were sectioned, stained with H&E staining that represents normal hepatic structure made up of healthy hepatic lobules and a central vein possessing radiating strands in untreated rats (Figure 6B). But an accumulated lipid content around the central vein and in between the hepatocytes as well as inflated and disrupted lobules were seen in obese rats (Figure 6C). Interestingly, the liver sections from obese rats treated with BSTN1 (Figure 6D) were seem to be better/equally recovered than that of Orlistat treated group (Figure 6E). However, central veins were found to be a little dilated in BSTN1 groups than normal but cells as such were found to be healthy.

### Effect of BSTN1 on Serum and Liver Lipid Profiles

The HFD-induced obese rats exhibited significant alterations in serum and liver lipid levels (over their respective control rats). Treatment with BSTN1 (5 mg/kg b. wt.) had significantly normalized the altered levels of serum total cholesterol (TC), triglycerides (TGL), high-density lipoprotein (HDL), low-density lipoprotein (LDL), very low-density lipoproteins (VLDL), phospholipids (PLs) and free fatty acids (FFA) (Table 2). Similarly, the liver tissue lipids including total cholesterol (TC), triglycerides (TGL) and free fatty acids (FFA) were also markedly normalized in the presence of BSTN1 (Table 3).

**TABLE 2:** Effect of BSTN1 on serum lipid profile of treated and untreated rats.

Parameters	Normal control	HFD control	BSTN1-1.25 mg	BSTN1-2.5 mg	BSTN1-5 mg	ORL-5 mg
TC (mg/dl)	84.0 ± 4.1	114.5 ± 13.2^#^	98.4 ± 9.4^*^	92.3 ± 9.3^**^	82.3 ± 14.3^***^	75.6 ± 15.3^***^
TGL (mg/dl)	148.9 ± 9.4	185.9 ± 8.7^#^	173.7 ± 6.4^*^	160.3 ± 6.6^**^	137.2 ± 8.6^***^	131.0 ± 9.2^***^
HDL (mg/dl)	45.8 ± 11.6	27.9 ± 10.5^#^	31.7 ± 12.9^*^	39.5 ± 13.5^**^	41.5 ± 10.1^***^	44.1 ± 13.9^***^
LDL (mg/dl)	62.8 ± 1.9	84.2 ± 1.7^#^	76.7 ± 1.3^*^	71.1 ± 1.3^**^	54.4 ± 1.7^***^	43.4 ± 1.8^***^
VLDL (mg/dl)	12.4 ± 15.8	29.4 ± 21.7^#^	21.0 ± 12.6^*^	17.8 ± 8.1^**^	13.4 ± 13.9^***^	9.0 ± 12.8^***^
PLs (mg/dl)	57.1 ± 3.2	112.7 ± 7.6^#^	101.4 ± 12.1^*^	84.3 ± 3.7^**^	63.4 ± 11.0^**^	61.7 ± 6.2^**^
FFA (mg/dl)	46.7 ± 2.2	96.3 ± 9.6^#^	87.4 ± 11.7^*^	64.1 ± 9.4^**^	50.5 ± 15.7^***^	44.7 ± 8.7^***^

The data is expressed as the mean ± SD, *n* = 6. ^#^ *p* < 0.001 indicates significant difference between HFD control and normal control. *, ** and *** indicate significant difference between HFD control and treated groups at *p* < 0.05, *p* < 0.01, and *p* < 0.001, respectively. TC, total cholesterol; TGL, triglycerides; HDL, high-density lipoprotein; LDL, low-density lipoprotein; VLDL, very low-density lipoprotein; PLs, phospholipids and FFA, free fatty acid.

**TABLE 3:** Effect of BSTN1 on hepatic lipid profile of treated and untreated rats.

Parameters	Normal control	HFD control	BSTN1-1.25 mg	BSTN1-2.5 mg	BSTN1-5 mg	ORL-5 mg
TC (mg/dl)	61.37 ± 5.3	118.2 ± 7.4^#^	111.6 ± 12.2^*^	91.3 ± 11.0^**^	67.6 ± 8.0^***^	62.9 ± 7.5^***^
TGL (mg/dl)	84.9 ± 2.7	156.1 ± 7.7^#^	127.7 ± 1.3^*^	97.8 ± 2.5^**^	79.07 ± 1.7^***^	81.6 ± 2.1^***^
FFA (mg/g)	69.34 ± 3.0	125.8 ± 2.3^#^	104.9 ± 3.0^*^	84.1 ± 2.3^**^	70.3 ± 2.7^***^	62.3 ± 2.1^***^

The data is expressed as the mean ± SD, *n* = 6. ^#^ *p* < 0.001 indicates significant difference between HFD control and normal control. *, ** and*** indicate significant difference between HFD control and treated groups at *p* < 0.05, *p* < 0.01, and *p* < 0.001, respectively. TC, total cholesterol; TGL, triglycerides and FFA, free fatty acid.

### Effect of BSTN1 on FBG, PPBG, F-Ins and HOMA-IR

Fasting blood glucose (FBG), post-prandial blood glucose (PPBG), fasting-insulin (F-Ins) and calculated HOMA-IR values were significantly diminished in BSTN1 treated rats when compared with HFD control rats in a dose dependent manner as shown in Table 4.

**TABLE 4:** Effect of BSTN1 on FBG, PPBG, F-Ins and HOMA-IR in treated and untreated rats.

Parameters	Normal control	HFD control	BSTN1-1.25 mg	BSTN1-2.5 mg	BSTN1-5 mg	ORL-5 mg
FBG (mg/dl)	86.12 ± 4.6	159.6 ± 8.0^#^	133.0 ± 6.1^*^	115.1 ± 9.2^**^	91.2 ± 7.1^***^	97.3 ± 6.9^***^
PPBG (mg/dl)	99.6 ± 5.0	211.1 ± 9.4^#^	174.6 ± 3.7^*^	141.5 ± 8.7^**^	100.3 ± 5.7^***^	104.7 ± 3.9^***^
F-Ins (µIU/ml)	14.57 ± 1.8	18.07 ± 1.3^#^	15.33 ± 2.1^*^	15.09 ± 2.0^**^	14.72 ± 1.7^***^	14.94 ± 1.1^***^
HOMA-IR	3.1	7.1^#^	5.0^*^	4.3^**^	3.3^***^	3.6^***^

^#^ *p* < 0.001 indicates significant difference between HFD control and normal control. *, ** and *** indicate significant difference between HFD control and treated groups at *p* < 0.05, *p* < 0.01, and *p* < 0.001, respectively. FBG, fasting blood glucose; PPBG, post-prandial blood glucose; F-Ins, fasting insulin; HOMA-IR, homeostasis model assessment index for insulin resistance.

### *In-Silico* Studies

**FIGURE 7:**
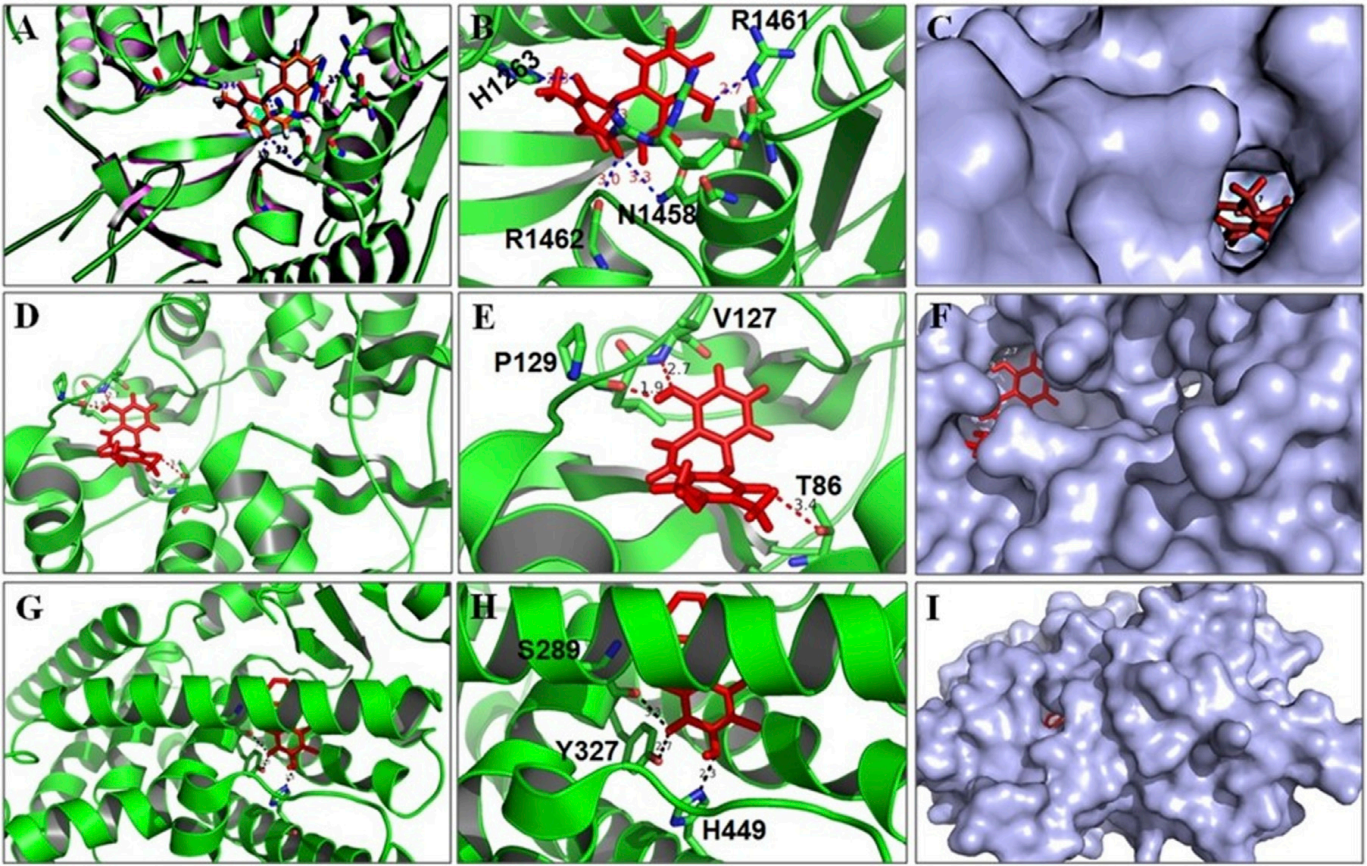
BSTN1 docking to FAS, AMPK and PPAR-γ. **(A−C)** Computational docking of BSTN1 (red) with the FAS (6NNA). **(D−F)** Computational docking of BSTN1 (red) with the amino acids of targeting AMPK (6C9F). **(G−I)** Computational docking reveals that BSTN1 (red) strongly interacts with hydroxyl and amino groups of the tyrosine, serine and histidine residues of PPAR-γ (3WMH). FAS, fatty acid synthase; AMPK, AMP-activated protein kinase and PPAR-γ, peroxisome proliferator-activated receptor-γ.

The outcome of the docking studies indicated that BSTN1 interacts with FAS (6NNA) with –100 binding energy. Consequently, the results showed that BSTN1 binds to FAS at the fatty acid binding site (substrate binding cleft), at amino acid residues His1263, Asn1458, Arg1461, and Arg1462 through multiple hydrogen (H) bonds. In addition, the site at which BSTN1 binds to the enzyme is very similar to that of the binding site for NADP and ethane diol, which suggests a stronger regulatory position for BSTN1 (Figures 7A–C). Docking studies predict that BSTN1 binds to AMPK by H-bonds close to the adenosine monophosphate (AMP) binding site with a binding energy of –148.62 (Figures 7D–F). But, close to the recognized inhibitor (JAA) binding location, BSTN1 interacts strongly with PPAR-γ with a binding energy of –194.0. Three H-bond interactions (–OH group of Tyrosine, –OH group of Serine, –NH group of Histidine) were observed between PPAR-γ and Bauhiniastatin-1 (Figures 7G–I) (Table 5).

**TABLE 5:** Molecular docking of BSTN1 (11687814) depicting ligand-protein binding interactions.

Ligand	Proteins	Binding energy	Bond length (Å)	Bonding interaction
Bauhiniastatin-1 (BSTN1)	PPAR-γ (3WMH)	–194.0	2.3–3	H-bond interaction [–OH group of tyrosine (Tyr327), JJA—OH group of serine (Ser289), –NH group of histidine (His449)]
	AMPK (6C9F)	–148.62	3–3.3	H-bond interaction (AMP binding site—Thr86, Val127 and Pro129)
	FAS (6NNA)	–100.0	3–3.6	H-bond interaction (His1263, Asn1458, Arg1461 and Arg1462)

## Discussion

Ready availability of energy dense foods and their frequent consumption coupled with sedentary life styles results in abnormal lipid profile, weight gain and metabolic disorders. Adipocyte biology reveals that, more and more accumulation of lipid droplets (triglycerides) in adipocytes leads to growth and expansion of adipose tissue to subcutaneous and visceral parts of the body (Jo et al., 2012). Diet-induced obese rats closely resemble human physiology and biochemistry and serves as an effective model for research and drug development in addition to *in-vitro* studies using 3T3-L1 cell lines (Rodríguez-Correa et al., 2020). Modern bioinformatic tools greatly contribute to elucidate molecule-ligand interactions and enables for effective drug development. Therefore, *in-silico*, *in-vitro* and *in-vivo* studies were conducted in this study to establish the anti-obesity efficacy of BSTN-1.

Maintenance of energy homeostasis in the body involves orchestrated play of complex biochemical and molecular events. At transcriptional level members of PPARs, C/EBPs and SREBPs play decisive role in adipocyte differentiation and lipid accumulation. The expression of these transcriptional factors is in turn regulated directly or indirectly by activated/inactivated AMPK. AMPK also plays a critical role in regulating hepatic lipid metabolism, glucose transport and gluconeogenesis. Hence, identifying therapeutic agents that target these molecules can serve as precious alternatives in the quest of anti-obesity drug development (Moseti et al., 2016; Agius et al., 2020).

In the *in-vitro* cell culture experiments, BSTN1 at 20 μM showed substantial reduction in lipid accumulation and adipogenesis in 3T3-L1 cells compared to untreated cells as evident from the Oil Red O stain and glycerol estimation assay (Figures 2A–C). Gene expression studies showed down-regulation of mRNA levels of PPARγ, SREBP1 and FAS in a dose dependent manner but AMPK expression remained unchanged in BSTN1 treated cells. This indicates, BSTN1 suppresses adipogenesis, lipogenesis and energy homeostasis by regulating at transcriptional level (Figures 3A–D). PPARγ consists of DNA binding domain, agonist-dependent and agonist-independent-activation domain expressed ubiquitously but abundantly in adipocytes. PPAR**γ**s play a central role in glucose homeostasis, fatty acid synthesis and adipogenesis (Grygiel-Gorniak, 2014). PPAR**γ** activates fatty acid uptake leading to neutral lipid accumulation in adipocytes which is sufficient for adipogenic induction making it a master regulator of adipogenesis (Shoucri et al., 2018).

In animal experiments, a substantial reduction in body weight gain and fat mass (abdominal fat), but no significant difference in food intake and water intake was noticed with BSTN1-treated rats over their HFD controls (Table 1). Biochemical assays of AST and ALT, serum and liver lipid profile indicate the beneficial role of BSTN1 to improve healthy lipid profile and hepatic function (Figure 6A and Tables 2, 3). In addition, fasting blood glucose (FBG), post-prandial blood glucose (PPBG), fasting insulin (F-Ins) and homeostasis model assessment index for insulin resistance (HOMA-IR) levels were diminished in BSTN1 treated obese rats in contrast to HFD controls (Table 4). This confirms a potential role for BSTN1 in attenuating insulin resistance and hence to be recognised as an anti-diabetic agent also. Certain key adipokines secreted by adipose tissue such as leptin and adiponectin are involved in regulating metabolic functions related to energy homeostasis, inflammation and adiposity. In the present context, oral administration of BSTN1 decreased leptin level but increased adiponectin level (Figure 5A). They might promote lipolysis, fatty acid oxidation and inhibit lipogenesis in liver and adipose tissue through AMPK activation. AMPK gets activated through phosphorylation of a conserved threonine in the activation loop of kinase domain in the α subunit or binding of AMP that can be mimicked by ADP and can be antagonized by ATP. Many of the AMPK activators including plant derived therapeutics like resveratrol and berberine activate AMPK by indirectly inhibiting ATP levels through increasing AMP and/or ADP (Srivastava et al., 2012; Kim et al., 2016).

Histological examination still remains an excellent technique to elucidate the ultrastructural modification of tissues. Liver and adipose tissue micrographs of BSTN1 treated groups (Figures 5, 6) evidently demonstrated their recouped architecture toward normalcy. These results support the alleviating effect of BSTN1 on serum and liver biochemical parameters mentioned above. Further, to confirm the therapeutic effect of BSTN1, western blot analysis was performed to observe the expression levels of PPAR-γ and AMPK in adipose and in hepatic tissues, wherein PPAR-γ was down-regulated while AMPK was activated (phosphorylated) (Figures 3, 4). This is in agreement with previous studies which showed activated AMPK diminishes the expression of C/EBPα, PPARγ, SREBP1 and acyl-CoA carboxylase (ACC) in adipose and hepatic tissues and thus inhibits synthesis of fatty acids and triglycerides (Ahmad et al., 2020). Some natural compounds such as capsaicin, guggulsterone, green tea extract, genistein and piperine were shown to regulate glucose homeostasis and fatty acid synthesis in liver as well as adipose tissue through PPARγ and AMPK mediated signaling to reduce fat accumulation (Feng et al., 2016). These results unambiguously emphasize the pharmacological efficiency of BSTN1 in alleviating obesity and improving hepatic function.

The molecular docking studies also suggest that BSTN1 binds AMPK at the AMP binding site through hydrogen bonding with Thr86, Val127, and Pro129. BSTN1 binding with Thr86 implicates that BSTN1 potentially activates AMPK function as well as expression through the interactions or vice versa. BSTN1 binds to PPAR-γ mediated by hydrogen bonding with amino acids Ser289, Tyr327, and His449. The observed down regulation of PPAR-γ expression could be in part due to these interactions initiating negative feedback loop. Our studies demonstrate that BSTN1 binds to AMPK near the adenosine monophosphate (AMP) binding site there by potentially mimicking AMP to activate AMPK. However, the binding of BSTN1 to the AMPK and PPAR-γ proteins seems to have positive and negative regulatory action on the transcription of AMPK and/or PPAR-γ respectively. In addition, potential binding prediction of BSTN1 with FAS indicates the inhibitory function of BSTN1 on FAS as well. BSTN1 binding at fatty acid binding site of FAS seem to be very interesting, but the lower negative energy of these interaction warrants to further investigate these enzyme substrate/inhibitor interactions (Figure 7). Previous studies reported that, 1w5-aminoimidazole-4-carboxamide riboside (AMPK activator) mediated activation of AMPK was shown to inhibit PPAR-γ and PPAR-α which may not be mediated through RXR or PPAR/RXR binding to DNA implicating potential multiple level regulation of BSTN1 on PPAR-γ (Sozio et al., 2011). This suggests complex agonist/antagonist binding dynamics with AMPK and PPAR-γ explaining BSTN1 binding to these two key proteins.

## Conclusion

Considering the clearly demonstrated results of *in-vitro* and *in-vivo* studies, an emphasizing pharmacological efficiency of BSTN1 against obesity and dyslipidemia is elucidated via PPAR-γ and AMPK modulation. These evidences are further complimented and confirmed by molecular docking studies. Therefore, BSTN1 could be considered as a potential pharmacological agent in drug development to combat obesity ailments.

## Data Availability

The original contributions presented in the study are included in the article/Supplementary Material, further inquiries can be directed to the corresponding authors.

## Abbreviations

ALT, alanine aminotransferase; AMPK, 5′ AMP-activated protein kinase; AST, aspartate transaminase; Bauhiniastatin-1, BSTN1; DMEM, Dulbecco’s Modified Eagle’s medium; FAS, fatty acid synthase; FBS, fetal bovine serum; HFD, High-fat diet; IBMX, 3-isobutyl-1-methyl- xanthine; MDI, differentiation medium of Induction; MTT, (3-(4,5-dimethylthiazol-2-yl)-2,5-diphenyl tetrazolium bromide); NAFLD, non-alcoholic fatty liver disease; PPAR-γ, peroxisome proliferator-activated receptor-γ; SREBP1, sterol regulatory element-binding protein 1.
